# Toward Advancing Nano-Object Count Metrology: A Best Practice Framework

**DOI:** 10.1289/ehp.1306957

**Published:** 2013-09-27

**Authors:** Scott C. Brown, Volodymyr Boyko, Greg Meyers, Matthias Voetz, Wendel Wohlleben

**Affiliations:** 1 Corporate Center for Analytical Sciences, DuPont Central Research and Development, Wilmington, Delaware, USA; 2 Material Physics, BASF SE, Ludwigshafen, Germany; 3 Core R&D–Analytical Sciences, The Dow Chemical Company, Midland, Michigan, USA; 4 Bayer Technology Services GmbH, Leverkusen, Germany

## Abstract

Background: A movement among international agencies and policy makers to classify industrial materials by their number content of sub–100-nm particles could have broad implications for the development of sustainable nanotechnologies.

Objectives: Here we highlight current particle size metrology challenges faced by the chemical industry due to these emerging number percent content thresholds, provide a suggested best-practice framework for nano-object identification, and identify research needs as a path forward.

Discussion: Harmonized methods for identifying nanomaterials by size and count for many real-world samples do not currently exist. Although particle size remains the sole discriminating factor for classifying a material as “nano,” inconsistencies in size metrology will continue to confound policy and decision making. Moreover, there are concerns that the casting of a wide net with still-unproven metrology methods may stifle the development and judicious implementation of sustainable nanotechnologies. Based on the current state of the art, we propose a tiered approach for evaluating materials. To enable future risk-based refinements of these emerging definitions, we recommend that this framework also be considered in environmental and human health research involving the implications of nanomaterials.

Conclusion: Substantial scientific scrutiny is needed in the area of nanomaterial metrology to establish best practices and to develop suitable methods before implementing definitions based solely on number percent nano-object content for regulatory purposes. Strong cooperation between industry, academia, and research institutions will be required to fully develop and implement detailed frameworks for nanomaterial identification with respect to emerging count-based metrics.

Citation: Brown SC, Boyko V, Meyers G, Voetz M, Wohlleben W. 2013. Toward advancing nano-object count metrology: a best practice framework. Environ Health Perspect 121:1282–1291; http://dx.doi.org/10.1289/ehp.1306957

## Introduction

The unique properties of nanomaterials that make them attractive for a plethora of applications, including their use in microelectronics, catalysts, composite materials, and biotechnologies, also invoke concerns for equally unique human and environmental risks associated with the use of these materials. It is not surprising that numerous governing bodies and policy makers around the world have invoked, or are considering invoking, definitions that specify what constitutes a nanomaterial for regulatory purposes. A concern within the chemical industry is that several of these definitions precede the current measurement science and concessions regarding the strict interpretation of the definition in addition to technological advancements are needed to enable practical metrology.

Our intent here is not to review the global state of definitions and policies, but rather to highlight current technology and knowledge gaps that are hindering advances in this area and to propose a tiered approach for moving forward. The European Commission (EC)–recommended definition of a nanomaterial (EC 2011a) is used as an important case example to highlight pertinent issues and challenges with regard to practical application of nano-object count–based metrics for categorizing materials as “nano” or “not nano.”

## The EC-adopted Definition of a Nanomaterial

*The definition.* On 18 October 2011, the EC recommended that nanomaterial be defined as comprising “natural, incidental or manufactured materials containing particles, in an unbound state or as an aggregate or as an agglomerate and where, for 50% or more of particles in the number size distribution, one or more external dimensions is in the size range 1 nm–100 nm,” where particles are defined as minute pieces of material with defined physical boundaries, an aggregate as a body of two or more particles that are strongly bound or fused together, and an agglomerate as a body of two or more particles that are weakly bound together by physical interactions (e.g., van der Waal forces) (EC 2011a). The application of volume-specific surface area (VSSA) [see Supplemental Material, “Volumetric Specific Surface Area (VSSA)—A Surface Area Approach,” p. 3] was also acknowledged as an agglomerate-tolerant proxy (Kreyling et al. 2010) to identify potential materials; however, number size distributions are to prevail (EC 2011a, 2011b).

The EC-adopted definition refines the International Organization for Standardization (ISO) definition of nanomaterialas exclusively applicable to materials consisting essentially of hard particles [solid nano-objects, defined as a material with one, two, or three external dimensions in the nanoscale (ISO 2010)], excluding solvated and self-assembled soft particles such as proteins and micelles as well as macroscopic nanostructured materials.

The EC-adopted definition is an attempt to create a uniform interpretation for identifying nanomaterials using particle size as the only metric, and it is specifically intended to classify a material as a “nanomaterial” for legislative and policy purposes in the European Union. The Scientific Committee on Emerging and Newly Identified Health Risks (SCENIHR; http://ec.europa.eu/health/scientific_committees/emerging/) has clearly expressed that classification as a nanomaterial does not imply that the material has a specific risk or new hazardous properties (EC 2011a, 2011b). Thus, the EC decided against a risk-based nanodefinition (Auffan et al. 2009) that would have addressed a much smaller number of materials based on other properties in addition to their size (Maynard 2011).

**Figure 1:**
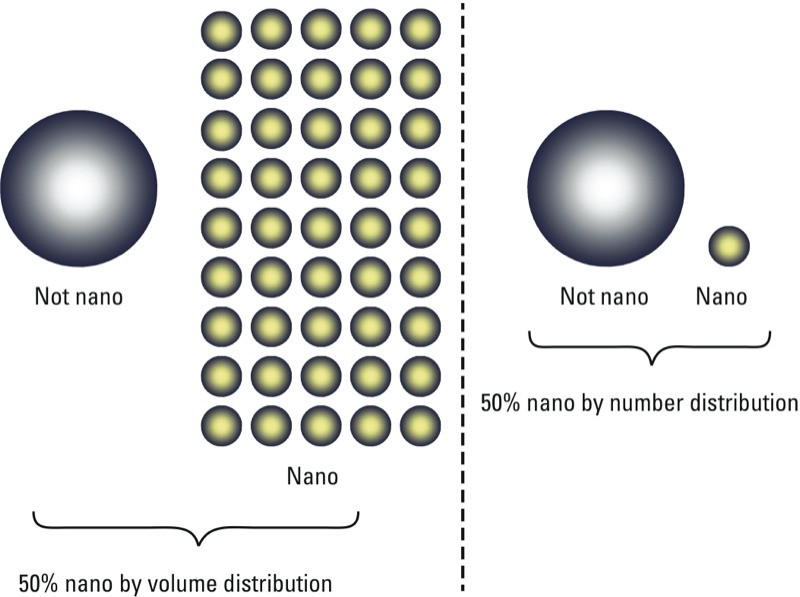
Schematic illustration of the difference between volume and number distributions and the EC-adopted definition.

*Challenges and implications*. The EC-adopted definition poses multiple challenges in the area of particle metrology (Appendix 1). Given the current state of nano-object metrology, any given technique [including electron microscopy (see Supplemental Material, “Scanning Electron Microscopy (SEM)” and “Transmission Electron Microscopy (TEM),” pp. 4–6)] may not be capable of accurately and efficiently identifying materials as “nano” or “not nano” based on the number percent requirement. This is largely due to the fact that current nano-object metrology standards have been focused mainly on mass and volume interpretations (Figure 1) and that associated round-robin exercises have typically employed relatively monodisperse materials that may not convey the complications associated with many real industrial materials (Lamberty et al. 2011). With the exception of electrical sensing zone measurements [see Supplemental Material, “Electrical Sensing Zone (Coulter Counting) & Scanning Ion Occlusion Spectroscopy (Ison),” p. 9] (conducted on micrometer-sized materials) and microscopy (conducted on model materials) (Allen 1997), there have not been substantial efforts to confirm the interexchangeability of number distributions with volume or mass distributions. Volume distributions and mass distributions provide representations of a population of particles wherein the particle size distribution is given in terms of particle volume or percent volume of particles for given size intervals or in terms of particle mass or percent mass of particles for given size intervals, respectively. Number distributions, on the other hand, are a representation of a population of particles wherein the particle size distribution is given in terms of particle counts or percent number of particles within the population for given size intervals. The interexchangeability of these metrics is largely dependent on the uncertainty of the applied technique as well as on inherent sample complexity.

**Figure 2:**
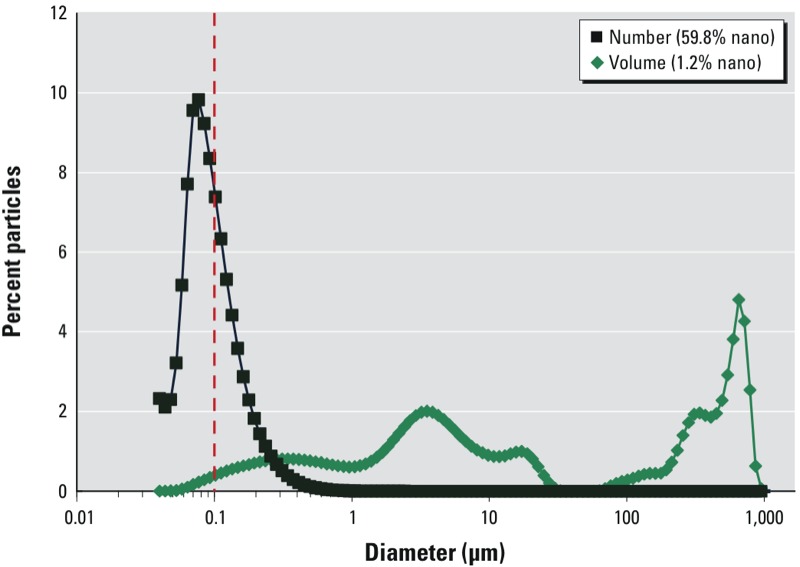
Laser diffraction and polarized intensity differential scattering determination of the volume distribution and calculated number distribution of a crushed chalk sample. For this broad distribution by volume, approximately 1% of material < 100 nm by volume accounts for > 50% of the material by number.

The lack of count-based nano-object standards and interlaboratory comparisons is a major concern because a 1% error in the ability of a method to accurately describe a mass or volume distribution at the nano scale could translate to a > 50% error in a number distribution (Figure 2). Furthermore, to our knowledge there have been limited efforts to validate particle count distributions that span < 100 nm, and such an assessment of the various techniques would be further hindered by the lack of nano-object count reference materials or instrumentation.

Therefore, at present, an absolute and universal method for nanomaterial determination with reference to the EC-adopted definition as well as other nano-object count– or number percent–based definitions does not exist. Without validated technologies or applicable reference materials, meaningful and equivalent methodologies for classifying materials based on particle number would be very difficult to develop.

Although the difficulties in applying modern metrology practices for determining particle size distributions were recognized as “challenging” by SCENIHR (EC 2011b), it is not clear whether the greater implications of not specifying a volume or mass percent number in addition to the current adopted limit of 50% by number was considered. The rationale for specifying a number percent rather than a volume percent or mass percent was to prevent a few large particles from skewing the populations of nanomaterials. However, at the same time the present definition may unintentionally and preferentially classify large materials as nano versus materials with a high particle content population in the vicinity of 100 nm. For instance, under the present definition, millimeter-sized ball bearings containing a minor amount of wear debris could be classified as nano, whereas a population of relatively monodisperse particles with a median diameter of 110 nm might not meet the EC-adopted definition requirements. The latter scenario would likely have a larger total surface area contribution from nanoscale objects as well as a larger total number of nanoscale objects and, therefore, potentially a higher likelihood of the material displaying nanosize-based properties. Similar inconsistencies that arise from the VSSA proxy were pointed out early in the consultation periods (Liden 2011). Furthermore, under the EC-adopted definition, the classification of materials may be further complicated by contamination issues. For instance, air contains a considerable number of natural or by-product airborne particles that are sub–100-nm aerosol particles (Table 1) (Penttinen et al. 2001a, 2001b; Ruuskanen et al. 2001; Stanier et al. 2004).

**Table 1:** Number concentration (nm/cm^3^) of particles in air collected at specified locations.

Location	Number concentration		Reference
	10–100 nm/cm^3^	100–500 nm/cm^3^	
Alkmaar, Netherlands	18,300	2,120	Ruuskanen et al. 2001
Erfurt, Germany	17,700	2,270	Ruuskanen et al. 2001
Helsinki, Finland	16,200	973	Ruuskanen et al. 2001
Pittsburgh, Pennsylvania, USA—urban	14,300	2,170	Stanier et al. 2004
Pittsburgh, Pennsylvania, USA—rural	6,500	1,900	Stanier et al. 2004

**Figure 3:**
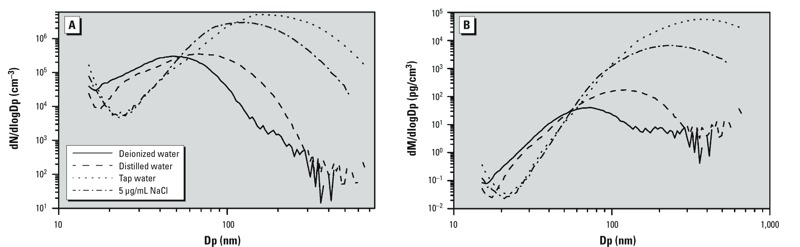
Number (*A*) and mass particle size (*B*) distributions determined for samples of deionized water, distilled water, tap water, and 5 μg/mL NaCl. Dp, particle diameter; NaCl, sodium chloride. Adapted from Knight and Petrucci (2003) and reprinted with permission from ACS Publications [*Analytical Chemistry.* Study of residual particle concentrations generated by the ultrasonic nebulization of deionized water stored in different container types, 75:4486–4492 (2003). Knight M, Petrucci GA. Copyright 2003 American Chemical Society].

Common water sources are also likely to be contaminated with low mass, but high number count, nano-objects (Figure 3). The same can be said for common vessels such as glass beakers and a variety of plastic containers used in laboratory analysis (Knight and Petrucci 2003). Under the current recommendation, many everyday materials would be classified as nano, which could inappropriately focus resources on a broad range of materials that were presumably not intentionally targeted by the EC definition. Clearly, there remains a need for refining the definition, including further appropriate specifiers to better address these issues. The inclusion of a specified volume percent over a limited size range (e.g., 1 nm–10 μm) and/or a total volume cutoff filter (e.g., a qualifying threshold or dust threshold) would largely enhance the identification of potential materials of interest and may also serve to simplify the associated metrology by enabling the application of more traditional metrology methods.

## Current Status and Knowledge Gaps in Nano-Object Count Metrology

*Overview of available metrology methods*. The vast majority of non–microscopy-based techniques for particle size measurement *a*) provide size distributions in terms of an equivalent spherical diameter that is an average, not a minimum dimension measurement, and *b*) interpret agglomerates and aggregates as being individual particles.

Therefore, for these methods to become suitable for classifying materials based on the EC-adopted definition, the material being tested needs to meet certain requirements. Namely, the sample must be composed of compositionally homogenous, mostly spheroidal or equiaxed particulates and be practically devoid of aggregates or agglomerates. From a product manufacturing point of view, these conditions may represent the exception rather than the rule. From a technical standpoint, this places a heavy burden on the development and application of appropriate and robust sample preparation systems to enable the use of both microscopic and non-microscopic particle size technologies with minimal error. Calzolai et al. (2012) recognized that “at the moment there is no single technique that can by itself provide a robust analytical method.” Furthermore, guidelines are needed to describe when one should and should not pursue characterization via microscopy and instead perhaps use a non-microscopic method. There are trade-offs for each approach, and expert judgment is required to maximize measurement accuracy while minimizing the resources and time needed for measurement.

Before a decision can be made regarding the best path forward, some form of microscopy (see Supplemental Material, “Imaging Particle Counting Techniques,” pp. 3–7) will be required to judge the practicality of measurement for at least microscopy, if not for multiple methods (Brown et al. 2010; Powers et al. 2006). For many samples, in particular those that consist of heterogeneous and irregularly shaped materials, a form of microscopy will likely be needed to satisfy the principles of the EC-adopted definition. That is, until reliable methods are developed to approximate the minimum dimension distributions in lieu of shape and in the presence of aggregates or agglomerates.

**Figure 4:**
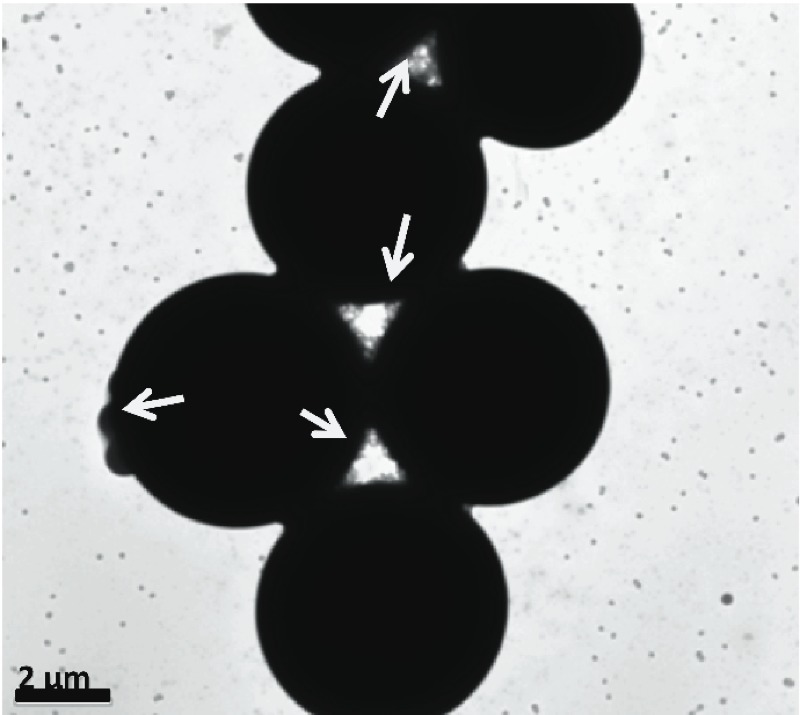
A mixture of nominal 6-μm, 220-nm, and 80-nm polymeric particle size standards. Note the accumulation of the nano and sub-micrometer particles underneath and at the interstices of the large micrometer-sized spheres via capillary assembly as indicated by the arrows. Without *a priori* knowledge of the sample, the interpretation of micrographs can be difficult because of numerous potential artifacts. TEM photomicrograph provided by C. Chan.

However, in situations where poor contrast (signal-to-noise) exists and background artifacts (due to, e.g., dispersants or other suspension constituents) are significant, microscopy methods may not be practical or even feasible. This would also be true in situations where population sampling and sample preparation artifacts become prevalent in microscopy, for instance, as for highly polydisperse samples where three-dimensional assemblies (Figure 4) may be unavoidable without advanced sample preparation techniques. This could also be an issue where chemically modified surfaces are used to constrain particles because the probability of attachment will also likely scale with size.

Several microscopy methods are capable of imaging nanomaterials with sufficient resolution. Based on availability and robustness, scanning electron microscopy (SEM) and transmission electron microscopy (TEM) are potential candidates for many materials. Atomic force microscopy (AFM) [see Supplemental Material, “Tapping Mode Atomic Force Microscopy (AFM),” pp. 6–7] is another viable option and would be the preferred form of microscopy for plate-like materials or those that tend to orient themselves on surfaces with their minimal dimension oriented upward. However, these methods also suffer from shortcomings, including counting inefficiencies, probe–sample interaction artifacts (e.g., electron beam degradation of the sample, probe-induced sample movement, artifacts due to electron beam inhomogeneity), sample preparation issues (e.g., sampling errors and debris artifacts related to sample preparation methods), and calibration and focusing errors. Therefore, data obtained via electron or probe microscopy may not be absolute for any given sample regardless of the measurement statistics.

Many real-world materials comprise broad constituent particle size distributions spanning several orders of magnitude. This is common for materials produced in part by size reduction processes, such as the simple example of crushed blackboard chalk presented in Figure 2. Difficulties in assessing particle populations via electron microscopy and combined approaches become pronounced due to statistical limitations and sampling when the material of subject has a broad range of particle sizes necessitating the use of multiple methods for microscopy (e.g., SEM, TEM, AFM, and light microscopy) because of each technique’s limitations in upper and lower magnification. Further, as Linsinger et al. (2012) noted, a suitable, commonly agreed characteristic external particle dimension must be defined for microscopy.

The presence of larger particles also increases the potential for sampling errors (Jillavenkatesa et al. 2001), may pose questions with regard to whether apparent nanoscale surface features are particulate in nature or not, and could serve to mask the presence of nanoscale objects by several mechanisms [e.g., capillary assembly beneath large particles, hiding them from view (Figure 4)]. Therefore, considering that both microscopic and non-microscopic approaches to nano-object count metrology are subject to errors, guidelines need to be developed to identify the best suitable method under a given scenario. Non-microscopic counting [e.g., electrospray ionization differential mobility analysis (ES-DMA) (see Supplemental Material, “Non-Imaging Particle Counting Techniques,” pp. 8–12)] and classifying techniques [e.g., asymmetric field flow fractionation (AFFF), centrifugal liquid sedimentation (CLS), analytical ultracentrifuge (AUC) (see Supplemental Material, “Classifying Techniques,” pp. 12–16)] are specifically recommended when microscopy may not be necessary or is not practical or, in some cases, not feasible. The determination of a particle count distribution by microscopy or other means may appear to be simple; however, it is actually a complex process with several levels of uncertainties that span from sample dispersion (i.e., the process of disassociating agglomerates within a sample into a population of constituent individual particles) and preparation to the measurement technique itself.

As in volumetric-based methods, no single particle count analysis technique is capable of spanning the full range of potential particle sizes (Allen 1997; Jillavenkatesa et al. 2001). Hence, multiple techniques will be required to span the full particle size range within a given sample. In many instances counting by microscopy may become prohibitively expensive, particularly for complex samples with low contrast and varied particle shapes. Hence, alternatives to manual microscopy methods are highly desired.

In terms of particle counting, few commercial methods are available for determining nano-object count distributions. Of these, perhaps only ES-DMA (Cledat et al. 2004; Guha et al. 2012; Jennerjohn et al. 2010; McEvoy et al. 2011; Pease 2012; Pease et al. 2009a, 2009b, 2010b; Tsai et al. 2010, 2011a, 2011b) is capable of counting particles from < 5 nm to approximately 1 μm in a manner largely independent of the material’s properties. Other available methods, such as commercial micro channel resonators (Burg et al. 2007; Lau et al. 2011) (Table 2) and nanoparticle tracking analysis (see Supplemental Material, “Non-Imaging Particle Counting Techniques,” pp. 8–12), struggle to count nano-objects below approximately 50 nm. The use of commercial micro channel resonators is limited by the relative density difference between the particle and the suspending media, whereas the use of nanoparticle tracking analysis is limited by the scattering efficiency of the material within the medium. Ideally, a method such as ES-DMA would be combined with a well-established macroscopic particle count method such as electrical sensing zone (Allen 1997; Barnard et al. 2012; Burg et al. 2007; Lau et al. 2011) (also known as Coulter counting) to cover the full range of material sizes likely present in the dispersion. The combination of ES-DMA and electrosensing zone methods would allow for a relatively inexpensive and commercially available method to count particles from approximately 3.5 nm to > 1 mm. Both of these methods can also be extended to evaluate minimum dimensions for nonspherical materials of consistent geometry (Baronas et al. 2007; Davies et al. 1975; Pease et al. 2009b). However, considerable method development, and perhaps instrument modification, may be required for materials that cannot be adequately dispersed in conductive fluid systems with appreciable vapor pressures, and the complete validation and round-robins are yet to be performed, with unknown outcome. Recent efforts in ISO technical committees [e.g., Technical Subcommittee on Particle Characterization (ISO/TC 24/SC 4) (2013)] are establishing standard protocols for nano-object count metrology using aerosol-based techniques involving DMA and associated counting methods (ISO 2009, 2013).

**Table 2:** Commercial non-imaging particle counting techniques.

Technique	Size range	Measured quantity	Limitations
ES-DMA/ES-SMPS	3.5 nm–1 μm	Aerodynamic electrical mobility	Requires conductive solutions, salts can confound results
Suspended microchannel resonator	~ 50 nm–several μm	Particle mass	Lower size highly dependant on density, microchannel fouling can lead to errors, requires calibration with particles of known mass
Scanning ion occlusion spectroscopy	~ 30 nm–several μm	Displaced volume	Particle membrane interactions can confound interpretation, shot noise, requires conductive liquid
Electrical sensing zone	200 nm–> 1 mm	Displaced volume	Requires conductive liquid, multiple apertures required to cover full range
Single particle optical sizing	200 nm–500 mm	Single particle turbidity	Independence of size and material properties not necessarily valid
Nanoparticle tracking analysis	10 nm^*a*^–1 μm	Single particle Brownian motion	Poorly scattering particle left out of analysis
Electrospray ionization mass spectroscopy–based methods	Varies substantially with method	Aerodynamic electrical mobility	Requires conductive solutions, salts can confound results, not routine, mass determined, size inferred assuming a density and shape.
SMPS, electrospray-scanning mobility particle sizer. ^*a*^The 10-nm lower limit is only possible with highly scattering materials (e.g., gold). (For additional information, see Supplemental Material, “Non-Imaging Particle Counting Techniques,” pp. 8–12.)			

When interpreting data from more common commercial particle size analysis techniques, care must be taken in extrapolating particle number distributions from methods that do not inherently count particles. Most modern particle size distribution equipment determine the approximate size of materials based on scientific principles linked to mass or volume fractions. Although one can generate a number distribution mathematically from a volume or mass distribution (Figure 2), the accuracy of this transformation is questionable because of errors in assumed apparent geometries, the accuracy of the initial methods (e.g., error multiplication), as well as the sensitivity of the chosen method to low concentrations of smaller materials. It is important to note that modern particle size metrology is largely a science of approximation wherein the generation of size distributions necessitates the inclusion of assumptions and fitting parameters that are not necessarily consistent from manufacturer to manufacturer or even within the same instrument under different analysis modes or parameters. These errors substantially increase as the material being analyzed increases in shape and size heterogeneity. Although some established techniques have been demonstrated to provide consistent measurements between mean size values in round-robin exercises [e.g., ASTM Standard E2490-09 (ASTM 2009)], the vast majority of these studies (including ASTM E2490-09) have utilized model materials that exhibit narrow size distributions. Many industrial materials have complex shapes and distributions that are not consistently sized by these methods unless the sample is segregated into a series of narrowly dispersed particle distributions.

Ensemble techniques (see Supplemental Material, “Ensemble Techniques,” pp. 16–19) such as dynamic light scattering (DLS) and acoustic attenuation spectroscopy (AAS) can largely under- or overestimate particle count distributions from measured volume/mass distributions because the width of distributions obtained by these methods is not necessarily representative of the true sample population. These methods are inherently of low resolution (compared with a particle count or classifying method). Specifically to the nanodefinition, a DLS polydispersity index of 0.1 was suggested as a suitable limit above which DLS data can no longer be interpreted accurately (Baalousha and Lead 2012). Although these methods are widely available and commonly applied, they are not expected to be suitable for accurately identifying number distributions from unknowns simply because they do not handle materials with complex distributions well. Although other ensemble methods such as low angle laser light scattering (e.g., laser diffraction) offer somewhat higher resolutions for larger materials, they also suffer from peak broadening artifacts often resulting in overestimated fine and coarse particle populations. Limitations in sensitivity and accuracy as well as signal-to-noise issues in the nanoregime further complicates interpretations. Ensemble methods tend to have excellent measurement reproducibility; however, they suffer from distribution inaccuracies that can exceed 50% for polydisperse materials (Allen 1997).

**Figure 5:**
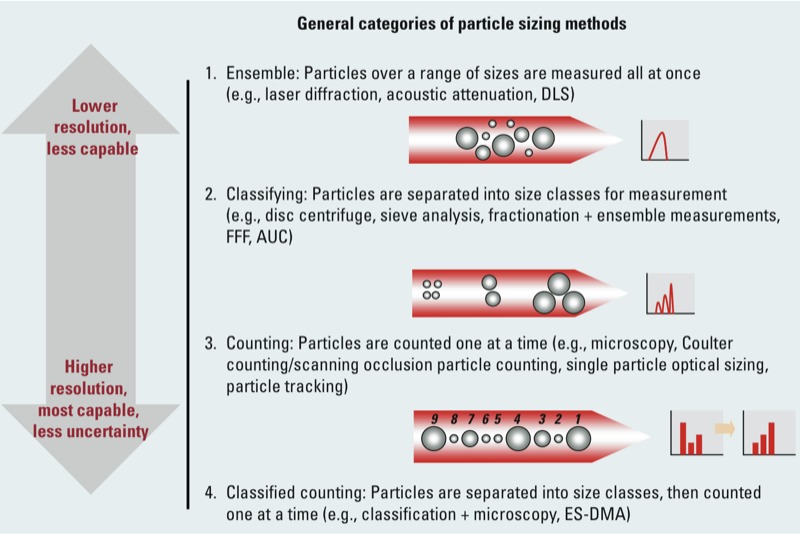
Schematic representation of the particle sizing method categories.

If counting methods are not available or practical for a particular material, classifying methods such as AUC or CLS and AFFF are anticipated to be suitable for a first approximation of nanomaterial content on a number basis. These methods apply physical forces for segregating or classifying particles by size in solution, enabling high-resolution particle size analysis (Figure 5). However, extreme care and highly skilled workers are required to properly apply these methods. McEvoy et al. (2011) recently demonstrated that AFFF combined with multi-angle light scattering (MALS) can determine viral particle number concentrations with an error of < 5%. Tsaiet al. (2011a) also independently demonstrated a linear correlation with AFFF-equipped MALS intensity and nanoparticle number counts generated by ES-DMA for 10- and 30-nm gold nanoparticles, as well as larger-sized agglomerates. However, the dynamic range of AFFF per condition is limited and multiple spacers, membranes, and run conditions would be required to access a dynamic range from a few nanometers to a few micrometers (von der Kammer et al. 2011). For larger materials, additional hyphenation or integration with other capable methods [e.g., sedimentation field flow fractionation (sdFFF)] will be required. Although the recommendation to consider AFFF is compatible with an earlier comparison of strengths and weaknesses of established techniques (Calzolai et al. 2012), the coupling of (several) detectors to a fractionation channel does not resolve the prerequisite of dispersion nor the errors of conversion to number metrics, which are small only for ideally dispersed spherical particles (Baalousha and Lead 2012).

For polydisperse substances, enhanced performance is expected for AUC (Wohlleben 2012) or, when sub–20-nm particle populations are not present, CLS. However, complications can arise in mixtures or coated materials because of potential density distributions. Corrections for these artifacts are available (Fielding et al. 2012) but require further evaluation. As particle shape becomes more heterogeneous and more complex, additional errors will occur in the number percent calculations from volume or mass distributions. Methods to get around some of these errors are presented in the literature (Allen 1997; Fielding et al. 2012). Academic approaches to classify materials on the basis of shape are far from routine and are not currently practical in an industrial setting.

Although classifying methods tend to be less reproducible than ensemble techniques, what they lack in precision is substantially made up for in the improved resolution and accuracy of the measurement. Although these methods are not fully validated replacements for counting techniques, they are anticipated to be a valid option when appropriate protocols are followed (Linsinger et al. 2012; Wohlleben 2012).

To our knowledge, it has been over a decade since the available commercial particle sizing instruments using largely different methods have been independently assessed and the results publicly reported (Allen 1997). Previously, assessments have historically dealt with materials consisting of easy-to-measure model reference material or real materials comprising continuous distributions. Few analyses have been conducted on particles of different shapes or for polydisperse systems. Even fewer analyses have been conducted to evaluate particle number distributions, and these have been confined mostly to micrometer-sized model particle systems. To our knowledge, no rigorous concerted interlaboratory evaluations of the ability of various techniques to accurately count industrial nano-objects have been completed. Moreover, nano-object count reference materials do not exist. Therefore, the ability of all techniques, including microscopic methods (i.e., SEM, TEM), need to be reevaluated to identify and mitigate artifacts. Some of these efforts are ongoing through recently initiated round-robin efforts [e.g., ISO Technical Committee 229, protocol development for primary particle size distribution by TEM, approved by resolution during the ISO/TC 229 Plenary meeting in Johannesburg (November 2011)]; however, additional research in this area is needed.

## Research Needs in Nano-Object Count Metrology

The current state of particle metrology is not readily equipped to address the definition of nanomaterial adopted by the European Commission. However, there are identifiable paths that likely will result in reasonable means to evaluate most materials. These paths, however, necessitate targeted research and advanced method development through the cooperation of industry, academia, government agencies, and instrument vendors. This concerted effort will be needed to arrive at affordable, accurate, and reproducible standard protocols for evaluating materials. Areas for research and development are identified below.

*Development and application of nano-object count reference materials.* The lack of nano-object count reference materials makes it difficult to evaluate the accuracy of the techniques employed. Both negative and positive nano-object count reference materials, or at least reference materials that should and should not fail the recommended European Commission criteria for a nanomaterial, should be identified and disseminated to ensure adequate refinement and reproducibility of the analysis methods. Materials with controlled deviations from a spherical shape must also be available as reference material.

*Methods to improve sample preparation for microscopy evaluation.* Sample preparation techniques for electron microscopy and AFM evaluation of the nanomaterials are critical to avoid sample bias as well as to reduce the number of images required to achieve a statistical count of particles. The actual number of particles that are required to be counted for statically relevant results depends on the particle distribution and can easily vary from < 100 to > 60,000 particles (Masuda and Iinoya 1971). The amount of time and number of images required to evaluate the upper limit of particles is currently not practical. Improved methods for sample preparation, such as electrospray deposition and other techniques that can increase the number of particles in a field of view without confounding the image analysis, are highly desired. Readily transferable and turnkey protocols for using these preparation methods are needed.

*Correlative nano-object size and chemistry.* Although the EC-adopted definition does not specify material composition as a metric, an understanding of correlated nano-object size and composition would be useful for risk assessment and process control activities. Rapid non-microscopic approaches to the identification of particle composition are desired to confirm that the sized materials are composed of the material in question. Hyphenated methods such as ES-DMA–inductively coupled plasma mass spectroscopy (ICP-MS) (Carazzone et al. 2008), ES-DMA–time of flight secondary ion mass spectroscopy (TOF-SIMS) (Fukuhara et al. 2008), and FFF–ICP-MS (Stolpe et al. 2005) have recently been applied; however, additional work is warranted.

**Figure 6:**
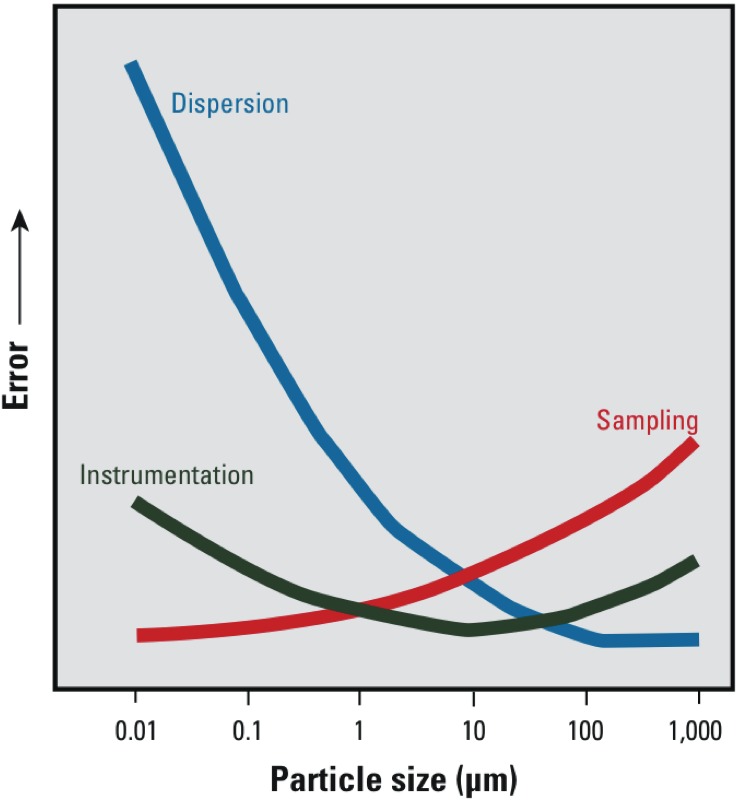
Sources of error in particle size analysis. Reprinted with kind permission from CSC Publishing [*Powder and Bulk Engineering*. Nanotechnology’s challenges = equipment manufacturers’ opportunities, 20:99–104 (2006). Moudgil BM, Brown SC, Krishna VB] and from Springer+Business Media: *Particle Size Measurements: Fundamentals, Practice, Quality*. Dispersion of powders in air and in liquids. Springer Particle Technology Series Vol 17. 1st ed. 2009, p. 119, Merkus HG, Figure 5.2. Copyright 2009 Springer Science+Business Media B.V.

*Advancements in dispersion science and methodology*. As particle size decreases, error in size analysis from inadequate dispersion increases (Figure 6) (Moudgil 2006). Although modern understanding of interfacial phenomena and energy transfer involved in dispersion has advanced significantly, it is still not possible to predict the conditions under which a material will be fully dispersed. Further advancements in this area are needed along with improved reporting requirements and procedures to enable adequate reproduction of dispersion between facilities and with differing equipment.

## A Tiered Approach toward Reasonable Nanomaterial Count Metrology

**Figure 7:**
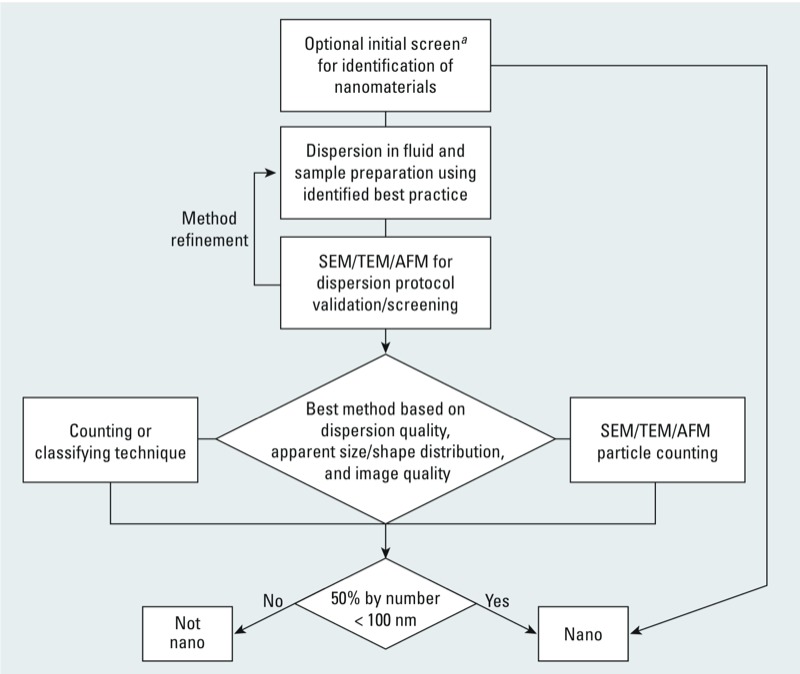
Proposed general characterization strategy for most industrial particulate products. ^*a*^Initial screening by ensemble methods (e.g., DLS, SLS, AAS) or VSSA measurement

*Overview*. Despite the challenges highlighted above, a tiered approach to addressing the characterization requirements is one way forward. This strategy combines multiple methods with the aim of simplifying the required measurements while highlighting what are believed to be the best available methods for the majority of materials. To clarify the proposed strategy, a decision tree outlining the approach is provided in Figure 7. This decision tree should be applicable to guide the selection of the best possible measurement as described in the EC recommendation Questions and Answers (EC 2011b), wherein it is stated, that the approach “should be an iterative process where practical experience will form an important aspect of the further development of methods and standards.” To enable future risk-based refinements to nanoparticle count–based definitions, this approach should also be considered in environmental and human health research involving the implications of nanomaterials.

*Manufacturer internal identification*. For a given material, the tiered approach begins with an optional initial screening to identify whether a material is clearly a nanomaterial. This step will exclude the use of unnecessary resources to scrutinize materials known to be nano by the manufacturer. As such the manufacturer or “company” decides upon the tools and dispersion protocols that may be applied for the initial analysis. The applied methods may include techniques and approaches that are not normally recommended for identifying a nanomaterial, including ensemble methods (e.g., DLS, laser diffraction, AAS) that are typically employed for process control purposes or even VSSA analysis. Even though the preferred techniques employed would consist of classifying or counting particle sizing techniques, these approaches may not be practical or necessary for defining materials that are clearly nano because of the limited availability of these methods and the advanced operator skill set required for their implementation. However, the measurement of particle number percent values of > 50% or VSSA values of > 60 m^2^/cm^3^ is not sufficient to classify a material as a nanomaterial without the written consent of the manufacturer. Ensemble methods and VSSA methods have severe limitations that prohibit their use for identifying materials as “nano” or “not nano.”

*Dispersion in fluid*. The next step, or the first step if the optional determination is deferred, is to disperse the subject material in fluid using an identified “best practice.” As particle size decreases, the influence of sample dispersion on the determined size distribution increases dramatically (Figure 6). In many systems, this is often due to intermolecular force scaling factors and associated phenomena (Israelachvili 2011). For instance, as particle size decreases, attractive van der Waals forces decrease scaled by the particle radius, whereas repulsive ion electrostatic forces decrease scaled by the radius squared. Hence, electrostatic stabilization of many nanomaterials can become impractical, and steric, electrosteric, or solvation (e.g., hydration force) interactions may be required to maintain stability (Israelachvili 2011). A considerable amount of energy transfer is often needed in order to disperse powdered samples into liquids. The efficiency of energy transfer processes for dispersion can change dramatically with the particle size of the same material. For instance, it may take 1 min of sonication to fully disperse a dried Stöber silica sample consisting of 500-nm particles with a narrow distribution, whereas it may take > 30 min to disperse a Stöber silica sample of dried 50-nm spheres, also of narrow distribution, using an identical sonication system and tip amplitude. Moreover, materials of varying surface composition experiencing different processing conditions can require largely different dispersion energies and routines. Many macroscopic materials such as crystallites and high aspect ratio material can also be reduced in size through high-energy processes; hence, a balance between chemistry and dispersion energy is often required.

The inclusion of dispersants such as surfactants and polymers will add additional challenges and sources of variation to already difficult particle counting analysis, whether performed by SEM, TEM, AFM, or ES-DMA. Van der Waals attraction can be reduced for many materials by selecting a suspending media of higher refractive index because the dominating contribution in van der Waals attraction typically is due to London dispersion forces (Israelachvili 2011). However, these liquids tend to have higher boiling points, lower vapor pressures, and high surface tensions, resulting in additional complications in the analysis through capillary assembly or via the presence of liquid films on the particles affecting, for instance, aerosol mobility measurements. Furthermore, direct aerosolization from powders or particulate films should not be overlooked. The use of modified matrix–assisted laser desorption methods may have merits for some nano-object systems in addition to classical shear-induced atomization methods. These complications are not trivial, but reasonable solutions likely already exist for many materials. There is, however, a need for information sharing and the identification of best practice dispersion protocols that are material specific yet adaptable to a wide variety of methods (Taurozzi et al. 2011). That versatility will require considerable method development: The first round of approaches for dispersion protocols may be specific to the analytical technique being applied. Experts from academia, industry, and government research facilities [e.g., the EC’s Joint Research Centre (Brussels, Belgium) or the U.S. National Institute of Standards and Technology (Gaithersburg, MD, USA)] may need to coordinate these efforts.

*Dispersion validation with EM*. A microscopic image will be required to validate the dispersion protocol. Directly viewing the sample will enable one to ascertain if milling or particle transformations other than dispersion in the media are taking place. Here it is important that the sample be prepared in a manner that inhibits capillary assembly or otherwise noninherent clustering of the material during preparation. Several methods exist for analyzing the dispersion of samples by SEM/TEM or AFM, but identification of agreed-upon best practices is lacking. The use of electrospray ionization and other emerging techniques (Hur and Won 2008; Jung et al. 2009; Lenggoro et al. 2002, 2006) for preparing non–close-packed samples are promising, in addition to more classical methods (Allen 1997; Jillavenkatesa et al. 2001). For highly polydisperse materials, the use of a Burt sampler (the slurry equivalent of a spinning riffler for powders) may be required, but sedimentation-induced bias could complicate analysis. Ideally, microscopic approaches will be integrated with size segregation approaches in the future as a synergistic hyphenated metrology technology to improve particle count accuracy and minimize the number of images and corresponding operator time required for analysis. Although some examples of this exist in the literature (Jung et al. 2010; Lenggoro et al. 2002; Li et al. 2011; Pease et al. 2009a, 2010a, 2010b, 2011; Suh et al. 2005), readily transferable methods and statistical interpretations are lacking.

The majority of materials will contain aggregates or agglomerates. Although revisiting the dispersion or sample preparation protocol for some materials and methods may result in significant enhancement of the quality of the sample, a threshold needs to be set for practical purposes. The standard methodology for identifying a dispersion end point is the determination of the point where particle size distributions plateau or do not significantly change with energy input (Taurozzi et al. 2011). Here, light scattering methods sensitive to larger particles are often useful for end point determination if more advanced methodologies such as classifying or ES-DMA are not practical or available. However, light scattering is likely not sensitive to relatively small changes in dispersion: CLS or other fractionating techniques are recommended if ES-DMA is not available. Nanomaterials must be sonicated carefully because bubble cavitation generates free radicals that can alter material properties, even leading to the creation or degradation of nanomaterials. The use of helium bubbling can significantly reduce the impact of sonication-induced free radicals by limiting the presence of dissolved nitrogen and oxygen species while limiting process modification and cost. Wear debris from the ultrasonic horn tip can also be a source of nano contaminants; hence, dispersion protocols must be developed with caution.

**Figure 8:**
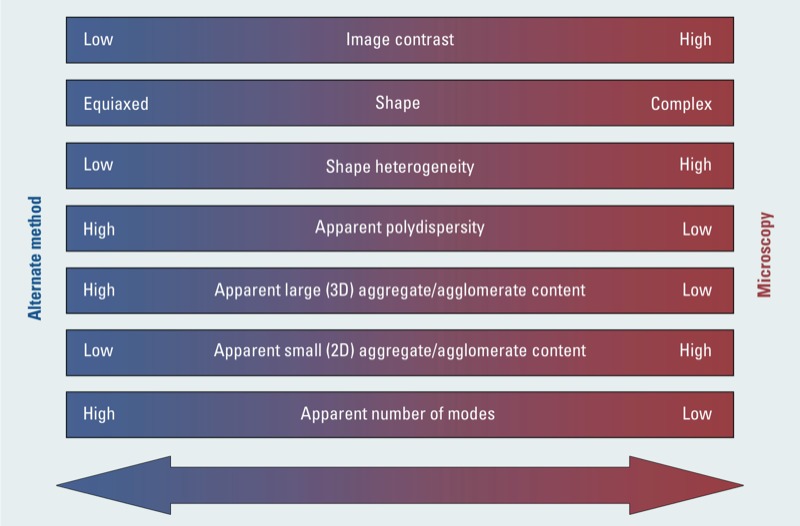
Selection criteria between methods to minimize error in particle count metrology.

*Expert judgment*. At full dispersion (loosely defined as the point of size distribution plateau, or where the particle size distribution remains flat with additional energy input), many real samples likely have aggregates or agglomerates present. Mandating that samples be devoid of aggregates to allow for non-microscopic techniques to be used is impractical. The overall objective of this review is to provide a reasonable approach for nanomaterial metrology in specific reference to the EC-adopted definition. Microscopy-based methods are not devoid of errors, and the best method must be determined from the intricate interplay of several factors. This involves considerations regarding the apparent shape and size distribution of the materials and complexity of the aggregates, the relative amount of contrast present, and apparent confounding issues such as the presence of residual dispersant films or sampling bias as determined by an initial SEM/TEM/AFM screen and complementary alternate analysis. In essence, a matrix must be developed to assess these issues. Figure 8 provides some considerations and a rough guide for method selection.

Using the guidelines should help determine whether a sample would be suitable for the imaging technique or would be better served by a non-microscopic method with the microscopy analysis performed during dispersion validation. The categories identified in Figure 8 attempt to capture the different sources of error stratified between microscopy and non-microscopic methods. Depending on the chosen method, additional variables such as the non-volatile solute content in the suspending medium will also need to be considered. More quantitative guidelines need to be developed as research into the magnitude and relative contribution of different sources of error in particle count metrology evolves. Each variable shown in Figure 8, with the exception of image contrast (the dominant consideration), is weighed equivalently. These guidelines suggest microscopy is the best choice for materials that exhibit high contrast, complex and heterogeneous shapes, low polydispersity, few three-dimensional particle clusters, considerable two-dimensional clusters, and constituent particle modal sizes. This selection process takes advantage of the unique capabilities in microscopy (i.e., the ability to handle complex and heterogeneous shapes) while minimizing the statistical number of particles that are required to be counted by promoting low polydispersity and few modal primary particle size populations and avoiding potential sampling issues (i.e., low three-dimensional aggregate content and polydispersity). At the same time, the selection process takes advantage of non-microscopic methods when particles are difficult to discern from the background (e.g., low relative contrast), have broad and multimodal distributions, and when reasonably equiaxed and uniform materials are present with few small aggregates. As a first approximation, the guidelines should point to the appropriate methods that should be used for analysis and will be later refined. Similar diagrams can be generated to assist in further narrowing the method selection.

In terms of microscopy, SEM and TEM are preferred; however, the advent of high speed AFM imaging technologies will likely promote AFM as an equally acceptable method for well-constrained materials deposited on atomically flat substrates. AFM currently stands as the preferred technique for materials such as obloids and plates that will likely align to substrates with their minimum dimension extended vertically.

The preferred alternate methods are ES-DMA + electrical sensing zone (ESZ), AUC, and AFFF. Count metrology via these techniques has been verified via TEM by multiple independent researchers (Lau et al. 2011; Lenggoro et al. 2002; Pease et al. 2009a, 2011; Tsai et al. 2011b); however, this selection process is not devoid of issues. Each method has advantages and disadvantages and may not be suitable for the given sample under all scenarios. For instance, ES-DMA + ESZ implies an as-yet unestablished derivation of the total particle number [as required by the EC recommendation Questions and Answers (EC 2011b)] and may not be valid for samples where a nonpolar solvent is required. Likewise, the use of AUC or AFFF is not suitable for effectively nondispersable powders and may not be suitable for samples that have complex and heterogenous shapes because of the required conversion to number percent (Calzolai et al. 2012; Linsinger et al. 2012; Wohlleben 2012).

## Conclusions

The increasing use of number percent population of materials as the defining metric for classifying particulate materials as nano or not brings new challenges in the field of particle metrology. In terms of volume or mass distributions, different techniques can generate answers with differences above the percent level. However, a 1% difference in volume distribution of nanoscale materials can exceed a 50% difference by number (calculated from the same distribution). Therefore, the validity of particle number and count distribution arrived at by different techniques (in particular techniques that measure volume- or mass-based parameters) need to be reevaluated in terms of identifying nanoscale material on a count basis. Electron microscopy and nano-object counting techniques are obvious methods of choice. However, a number of artifacts are linked with these techniques.

Existing and emerging nano-object counting particle sizing techniques have yet to be vetted and have sample preparation limitations. Advanced classifying methods, such as FFF and AUC, may provide additional solutions to accurate metrology in this size range, but they are not validated for the specific purpose. To improve analysis throughput and accuracy, the development of hybrid or hyphenated classifying and microscopic techniques and advanced sample preparation methods for evaluating nanomaterial content is desired, but not yet practical.

Based on the current state of the art, we propose a tiered approach for evaluating materials. Best practices for dispersion and sample preparation in the tiered approach should be developed in strong cooperation between industry, academia, and national research facilities and concerned associations such as the Cefic European Chemical Industry Council (http://www.cefic.org) and producers of possible nanomaterials. There is a clear need for targeted research in the area of nano-object count metrology to adequately address the recommended definition of nanomaterial proposed by the European Commission. This will require concerted efforts from industry, academia, and regulatory agencies as well as instrument vendors.

Substantial scientific scrutiny is needed in the area of nanomaterial metrology to establish best practices and to develop suitable methods before implementation of the adopted number percent definitions for regulatory purposes. We have suggested areas for improvement. We also recommend further specification of future definitions to include a volume or mass basis number (likely defined by technique and potentially device) and potentially a risk-based metric.

The analytical challenges associated with nano-object number specifications are rooted in the nontraditional analyses required to fulfill this metric for the purpose of nano classification. For the EC-adopted definition example, the recommended definition necessitates *a*) the determination of the minimum external dimension number distributions for a representative sample of a given material, and *b*) the counting of constituent particles within aggregates or agglomerates as individual particles. The reliability of current analytical techniques to accurately meet both of these requirements is not fully understood. The casting of a wide net with still unproven metrology methods may stifle the development of emerging and sustainable nanotechnologies.

## Appendix

**Appendix 1:** Characterization Challenges Posed by the European Commission Recommendation on the Definition of Nanomaterial^*a*^

Challenge	Explanation
The definition implicitly mandates that constituent particles within aggregates be counted.	Most modern techniques for particle size determination size aggregates and agglomerates as if they were constituent particles. The use of such methods will need to be validated on a case-by-case basis and will depend on sample form and sample properties.
The definition is based on a minimum external dimension rather than an average dimension.	As materials move from mostly rounded shapes to shapes with complicated geometries (often the case for industrial products), the determination of a minimum dimension becomes difficult, especially by transmission electron microscopy (TEM), which is inherently a two-dimensional technique.
The definition specifies a 50% by-number distribution.	Techniques for particle counting in the nano-range are limited. Currently there are no particle count reference materials in the nano-range, making it difficult to cross-correlate and validate methods. In particular, the accuracy of converting a mass- or volume-based measurement distribution to a number distribution is questionable.
^***a***^EC (2011a, 2011b).	

## Supplemental Material

(291 KB) PDF
