# Supplementary material for: Toward Advancing Nano-Object Count Metrology: A Best Practice Framework
Source: Environ Health Perspect. 2013 Sep 27;121(11-12):1282–91. doi: 10.1289/ehp.1306957 (PMC3852792; doi:10.1289/ehp.1306957)
Supplement: (291 KB) PDF [file ehp.1306957.s001.508.pdf]

Supplemental Material

Toward Advancing Nano-Object Count Metrology: A Best Practice Framework

Scott C. Brown,[1] Volodymyr Boyko,[2] Greg Meyers,[3] Matthias Voetz,[4] and Wendel Wohlleben[2]

[1]DuPont Central Research and Development, Corporate Center for Analytical Sciences, Wilmington, Delaware, USA

[2]BASF SE, Material Physics, Ludwigshafen, Germany

[3]The Dow Chemical Company, Core R&D-Analytical Sciences, Midland, Michigan, USA

[4]Bayer Technology Services GmbH, Leverkusen, Germany

Corresponding Author:

Scott C. Brown

DuPont Central Research & Development

Experimental Station 323/110A

Route 141 & Henry Clay

Wilmington, DE 19803 USA

Scott.C.Brown@usa.dupont.com

(302) 695-3532

Table of Contents

Volumetric Specific Surface Area (VSSA)–A Surface Area Approach 3
Particle Sizing Methods 3
Imaging Particle Counting Techniques 3
Transmission Electron Microscopy (TEM) 4
Scanning Electron Microscopy (SEM) 5
Tapping Mode Atomic force Microscopy (AFM) 6
Optical Ultramicroscopy 7
Non-Imaging Particle Counting Techniques 8
Suspended Microcantilever Resonator (SMR) 8
Electrical Sensing Zone (Coulter Counting) & Scanning Ion Occlusion Spectroscopy 9
Dynamic Mobility Analysis (DMA) and Electrospray-Dynamic Mobility Analysis (ES-DMA) 9
Nanoparticle Tracking Analysis (NTA) 11
Classifying Techniques 12
Cascade Impactor 13
Field Flow Fractionation 14
Analytical Ultracentrifugation (AUC) and Centrifugal Liquid Sedimentation (CLS) 15
Hydrodynamic Chromatography 15
Ensemble Techniques 16
Small Angle X-ray/Neutron Scattering. 16
Ultrasonic Attenuation Spectroscopy 17
Laser diffraction spectroscopy 18
Dynamic Light Scattering (DLS) and Similar Techniques 18
References 20

Volumetric Specific Surface Area (VSSA)–A Surface Area Approach

The volume specific surface area (VSSA) requires measurement of both the mass specific surface area and density. These are readily measured using BET (Brunauer, Emmett and Teller) gas adsorption (Allen 1997; ISO 2010) and Helium pycnometry (Allen 1997), respectively. Although VSSA determination cannot be used to demonstrate that a material is not a nanomaterial as specifically noted by the definition set forth from the European Commission (EC 2011), it can be used to identify a material as a nanomaterial for densified materials of homogenous composition as deemed by the manufacturer. VSSA measurement is not a replacement for particle count metrology as many heterogenous and macroscopic porous materials may have high VSSA without being 'nano' (e.g., several pore-size distribution certified reference materials available from NIST or BAM) according to the EC adopted definition. In principle, the threshold that is applied for the application of VSSA is a value is greater than 60 m^2/cm^3, at or above which a material is considered to likely be nano. This arbitrary value—identified in the definition—corresponds to the theoretical VSSA of a population of monodisperse 100nm spheres. For products like fumed silica, VSSA measurement offers a simple an inexpensive route to identify densified materials of homogeneous composition as 'nano' if this metric is deemed to be acceptable by the manufacturer.

Particle Sizing Methods

Imaging Particle Counting Techniques

Imaging or microscopy methods are anticipated to be suitable for classification purposes in the absence of confounding artifacts when sufficient resolution and contrast are present. These

methods are subject to significant sample preparation errors. Round robins are required to establish uniform sample preparation and analysis methods specifically for the purpose of nanomaterial classification.

Transmission Electron Microscopy (TEM)

- Size range: 0.5nm – 10µm; Resolution: 0.2nm; typical concentration: mg-µg; direct particle counting method; geometric diameter

TEM is a direct imaging technique that uses a magnetically focused electron beam (100-300keV) to image samples based on electron scattering and absorption contributions to loss of transmitted electrons. The image contrast is determined primarily by electron density, hence atomic number, where scattering is less for low atomic number materials and higher for high atomic number materials. In modern TEMS image formation occurs on a CCD array inside a digital camera. Since the wavelength of an electron (DeBroglie wavelength) is small, the resolution of a TEM is capable of atomic resolution. Further, to minimize electron-molecule collisions, the microscope operates in high vacuum. This is an important consideration for sizing materials that might change size or shape due to removal of volatile material or structural collapse. The former may be controlled using cryogenic sample holders. Sample preparation requires dispersion of nanoparticles onto TEM grids with supporting films (e.g. carbon) from dilute solution or aerosol spray. Negative contrast staining may be employed to enhance the edges of nanoparticles. TEM can image particles consisting of atom clusters (~0.5nm) up to thousands of nm. Since the image is a 2D projection the particles may be sized based on lateral measurements obtained directly from the image scaling. The digital camera magnification is often calibrated using the lattice spacing obtained from gold nanoparticles or carbon nanotubes. Resolution is calculated from the contrast transfer function of the microscope. With digital

image capture, image analysis can be automated if contrast is good using digital image processing software. Since the imaging is direct, the sizing information can include equivalent diameters or other shape related metrics. TEM also can provide information on 3D shape using rotatable holders that can provide images through many angles that can be put together to provide tomographic information. Compositional information may be obtained in spectroscopic modes such using energy dispersive detection of electron induced x-ray fluorescence or by electron energy loss spectroscopy.

Scanning Electron Microscopy (SEM)

- Size range: 3nm – mm; Resolution: 1nm; typical concentration: mg-µg; direct particle counting method; geometric diameter

SEM is an imaging technique which uses a focused primary electron beam (500eV to 30keV) to raster scan the surface of a solid. At these energies the beam the penetration depth of the electron beam is only a few microns or less. The beam-solid interaction results in the formation of low-energy secondary electrons (SE signal, surface sensitive), backscattered electrons (BSE signal, higher energy from deeper in the solid), and electron induced x-ray fluorescence (XRF signal, compositional information). To minimize charging effects for secondary electron imaging non-conductive samples must be coated with a metal by sputtering or evaporation. The SEM can be operated in low vacuum or environmental imaging using an imaging gas (typically water vapor) to control charging (without metal coating) and/or to control swelling of hydrophilic nanomaterials. Liquid or wet cells and cryogenic stages are now available for SEMs. Modern field emission sources provide ready visualization down to about 30nm, e.g. to see a 30nm particle within and aggregate. For dispersed particles reliable sizing down to 10nm is possible. The SE or BSE image is a 2D projection and the particles may be sized based on lateral

measurements obtained directly from the image scaling and instrument calibration. With digital image capture, image analysis can be automated if contrast is good using digital image processing software. Since the imaging is direct, the sizing information can include equivalent diameters or other shape related metrics. Particle size distribution is based on counting statistics obtained from analysis of several images in order to be representative of the population.

Tapping Mode Atomic force Microscopy (AFM)

- Size range: 1nm – 5000nm; Resolution: 0.1nm; typical concentration: mg-ug; direct particle counting method; geometric diameter from height measurement

AFM is a direct imaging technique that relies on mechanical contact between a nanoscale tip and the nanoparticles deposited on a surface. The most common methods of AFM are dynamic, where the tip is attached to a cantilever that is driven at resonance such that the tip oscillates in a controlled manner and can be brought into a very gentle tapping or intermittent contact with the surface while scanning. The size of a nanoparticle measured in this way is most accurate for its height since only at the highest point on the particle does the tip apex touch. The lateral dimensions of the particle can generally not be used for size due to mixing of the tip shape with the particle shape, especially when the tips have typical radii of 5-10 nm, which is of the same order as the particles being measured. For incompressible particles AFM can measure sizes from 1 nm to 5 um over a lateral range of 100um x 100um on a surface. Measurements can be made in atmosphere, fluid, or vacuum. The particles must be attached well to the surface or they will move under the influence of the scanning tip. The technique is well suited to spherical, rod, or plate particle shapes. Irregular or faceted structures will be problematic. To achieve reasonable counting statistics several hundred particles should be counted. This means that data from several images will have to be combined to characterize the particle size distribution. A high

resolution Tapping mode AFM image can be acquired in about 10 minutes although recent developments with fast scanning systems should reduce this time by an order of magnitude. Since the method relies on measurement of height, the AFM can be calibrated using traceable step height standards from various vendors. Particle heights can be readily assessed using post-processing and image analysis algorithms that generate height histograms. Systems with closed loop z-sensors are more expensive and will be most accurate for height measurement but also are slightly noisier than open loop counterparts. Both types require calibration. Available Standard: ASTM E2859-11 (ASTM 2011).

Optical Ultramicroscopy

- Size range: 50nm – 10000nm; Detection to 10 nm; typical concentration: 10^6-10^{10} particles/mL; direct particle imaging method; diameter of diffraction ring

Recent developments in darkfield optics have shown improved resolution (lambda/5) for non-diffraction limited measurement. This has been achieved using a cardioid annular condenser that replaces a conventional condenser in a transmitted light microscope, effectively providing a super resolution dark field image with features that can be resolved below 100nm (Vainrub et al. 2006). Proper alignment of the optical components and optimization of the illumination on the condenser, exposure time, and NA of the lens are critical parameters to achieve highest resolution. With respect to particle sizing the technique relies on collection of size standards of similar composition (optical properties) of the materials to be tested. The darkfield intensity has been found to correlate with particle size for polystyrene standards as small as 100 nm (Rothe et al. 2007). A number of other super resolution microscopy methods are also available [e.g., stimulated emission depletion (STED)] and are reviewed elsewhere (Huang et al. 2009).

Non-Imaging Particle Counting Techniques

Particle counting techniques size particles one at a time. Methods for nanoparticle counting are currently classified as emerging particle size analysis techniques. These methods are anticipated to be suitable for nanomaterial classification; however, concerted round robin efforts are needed.

Suspended Microcantilever Resonator (SMR)

- Size range: 50nm – 3um; Resolution: 10^{-15}g; typical concentration: 10^7 particles/mL; direct particle counting method; mass measurement

The resonant microcantilever technology is based on the frequency shift of a hollow, resonating microcantilever as individual nanoparticles are conveyed through the free end in fluid (Burg et al. 2007). The cantilever is hermitically sealed in vacuum in order to increase its quality factor. The NPs are conveyed through the lever using a microfluidics gating system which samples from dilute solution. As with commercial AFMs, the resonance frequency of the cantilever is monitored using a laser detection scheme. The frequency shift comes from the added buoyant mass the cantilever experiences when a particle travels through. The buoyant mass can be related to a dry mass through the density of the conveying fluid, usually water, and the particle density. The channel dimensions within the cantilever are typically 8 micron x 8 micron or smaller, so the system has an upper limit on particle size. The lower detection limit on size is primarily dictated by the density of the nanoparticle, so the technique will favor metals and dense inorganics over carbonaceous, polymeric, or biological materials. The lower mass resolution for commercial systems is about a femtogram, which would include, for example, a 50 nm diameter gold particle but exclude a 50 nm SiO_2 particle. The technique is a counting technique and sampling can be done at a reasonable rate, about 1 Hz so that >1000 particles can be measured in

about 20 minutes. Particle size distributions are represented directly as mass distributions that can be converted to equivalent diameter or shape distributions. This technique can be used to measure the density of nanoparticles by making measurements in two different fluids with different densities.

Electrical Sensing Zone (Coulter Counting) & Scanning Ion Occlusion Spectroscopy (Izon)

- Size Range: 50nm- 1mm; typical concentration: ppt – ppm; volume measurement; equivalent volume spherical diameter

The electrical sensing zone method is an established particle counting method that measure the volume of electrolyte displaced by a particle as it passes through an orifice via a differential impedance measurement. The traditional form of this method applies the use of fixed apertures and is capable of sizing materials from over 1mm to just 200nm (using multiple apertures). Through the use of a flexible and stretchable aperture technology, a commercial system is now available that enables the measurement of particles from 50nm to 10 microns. In both of these methods, errors originate from particle interactions with the aperture walls, and the minimum size is often limited by the Schott Noise. Conductive particles also pose analysis problems. Available Standards: ISO 13319:2007 (ISO 2007a), ASTM C690-09 (ASTM 2009a).

Dynamic Mobility Analysis (DMA) and Electrospray-Dynamic Mobility Analysis (ES-DMA) (Variants include Scanning Mobility Particle Sizing (SMPS), Ion Mobility Spectrometry (IMS) and Gas Phase Electrophoretic Mobility Macromolecular Analysis (GEMMA) and Electrical Aerosol Analyzer (EAA).

- Size Range: 2.5nm-1000nm; Resolution: ~2nm;typical concentration: ppt – ppm; Particle count measurement; aerodynamic electrical mobility

DMA is a particle classification method that selects nano-sized particles base on the balance of drag and electrical forces at atmospheric pressure. A key feature of DMA is that the particles entering the unit are neutralized with a bipolar charger such that a Boltzman distribution of particles with positive or negative singlet charge primarily exist. Neutralization typically occurs through the use of a radioactive sealed source (e.g., Kr-85 or Po-210) or through the use of soft xrays. Once entering the DMA, only positively or negatively charged particles are then classifyied using an oppositely charged bias in the DMA. DMAs are designed to exhaust only "monodisperse" populations of particles based on the applied voltage and applied sheath flow. This is assuming the population of particles only consists of singlet charges; however corrections for multiply charged particles are available. These size-selected particles are then typically counted by a condensation particle counter (CPC) or an electrometer. The use of a DMA in conjuction with CPC in a continuous scan mode is distinguished as SMPS; however, in the literature and within this review DMA and SMPS are interchangeable since the emphasis is often on the general technique rather than the specific mode of operation. In this light DMA and SMPS as well as IMS, GEMMA, and EAA are essentially equivalent. The technique is capable of classifying and counting particles from approximately 2nm to 1000nm. It should be noted that the counting efficiency of classified materials can depend significantly on the design of the applied CPC and the liquid applied (typically butanol or water). ISO/DIS 27891 (ISO 2013a) provides guidelines for the calibration of condensation particle counters. ISO 15900 (ISO 2009a) provides guidelines for determination of aerosol particle size distribution by means of the analysis of the electrical mobility of aerosol particles.

ES-DMA is a modification of the traditional DMA system wherein an electrospray ionization source (ESI) is added to generate aerosols from liquid suspensions extending the SMPS method

to a wide range of colloidal suspensions. ESI aerosolizes the suspension through the use of a high voltage electric field that causes the liquid suspension to eject from a developed Taylor cone the end of a metal needle. Upon ejection the droplets further disintegrate via Rayleigh breakup. Through this process the particles suspended in the liquid phase are re-generated as aerosols that are then neutralized to a bipolar charge distribution and analyzed as in DMA. This method is actively being developed (Allmaier et al. 2008; Guha et al. 2011; Guha et al. 2012). Electrospray systems for commercial ES-DMA devices have been designed for ~sub-200nm particles and may pose difficulties for larger samples without further modification. This method requires that the suspending fluid be conductive and also have an appreciable vapor pressure, hence applicable fluids and salts can be limited. However, since exhaust particles are extractable and can be subsequently imaged via TEM and characterized by other means, appropriate corrections can be made for a wide range of situations. Several methods have been published for this method; however, an applicable standard protocol has yet to be developed for ES-DMA. Available Standards: ISO 15900 (ISO 2009a), ISO/DIS 27891 (ISO 2013a).

Nanoparticle Tracking Analysis (NTA)

- Size range: 10nm – 600nm; typical concentration: 10^6-10^{10} particles/mL; particle tracking method; diffusion measurement providing hydrodynamic radius

NTA combines an optical microscopy cell with a laser tracking system to measure the diffusion of particles in a liquid due to Brownian motion. Particles are ‘visualized’ due to scattered laser light from particles within the 80um wide focused beam that is in close proximity and parallel to a highly polished aluminum plate within the cell. The particle motion is tracked dynamically using a CCD camera with time resolution of about 30 frames per second. Particle motion is sampled over 10s of seconds. The scattering is use solely to track position and not size.

Particles can be tracking by comparing position on a frame-by-frame basis using particle tracking software where the mean squared displacements are tracked. Since the particles are moving in 3 dimensions they may only be tracked within the focal range of the optics so criteria must be established for collection time. This could lead to some bias in polydispered systems. From the trajectory analysis the diffusion coefficient can be determined and, therefore, the spherical equivalent hydrodynamic radius using a modified Stokes-Einstein equation that accounts for three-dimensional motion. Since every particle is tracked it is possible to generate profiles that reflect the number count. NTA may have difficulty with agglomeration since the agglomerate will appear as a single scattering center. In these cases the sample dispersion may have to be altered through further dilution or sonication. Dispersing aids may be used but should not index match the particles. Larger particles in a population may be removed through filtration, centrifugation or settling prior to analysis. Errors also arise as the particle shape deviates from a sphere since the existence of multiple diffusion coefficients are not considered in the analysis. It should be noted that NTA system relatively new and has not been fully vetted. Commercial devices are also currently available through only two vendors and sample analysis outcome can be highly dependent on user-defined variables. Available Standards: ASTM E2834-12 (ASTM 2012).

Classifying Techniques

Classifying techniques employ methods to physically separate particles by size prior to detection and population analysis. These methods are typically sensitive to small fractions of particles, by virtue of the separation process. Many classifying techniques are anticipated to be suitable for

the purpose of classifying nanomaterials. As with particle counting techniques, round robins are needed to access uniformity in nanomaterial classification.

Cascade Impactor

- Size range: 10nm – 20000nm; typical concentration: mg-ug; classifying particle method; mass separation based aerodynamic diameter

Cascade impactors are classifying techniques that utilize inertial separation of solid or liquid particles in a gas phase (e.g. air) to separate particles based on an aerodynamic diameter. Aerosolized particles are forced through a series of stages each containing an impaction plate and each having smaller and smaller jet orifices to pass through to the next stage. Each stage has a characteristic cut-off size. Nanoscale separation is enhanced when jet expansion is done at lower than ambient pressure such that the particle sizes are less than the mean free path of air molecules making up the carrier gas. The characteristic cut–offs that are progressively smaller due to higher acceleration through progressively smaller nozzles. The aerodynamic diameter is the diameter of a sphere of unit density (1g/cc) that has the same gravitational settling velocity as the particle. At each stage particles can be collected or trapped onto solid filter supports (e.g. PTFE, polycarbonate, or aluminum) mounted on to the impaction plate for subsequent analysis for size (microscopy), weight (gravimetric), or composition (e.g. XRF, TGA/MS, ICP-MS). Cascade impactors are commonly used for aerosol research and inhalation studies. Typically the sizing is done by mass fraction of recovered particles obtained at each stage, i.e. the mass fraction is binned according to the cut-off size. Total recovery of the particle size distribution is difficult using impaction approaches since more than 10 stages may be used in series. Also impactors suffer from stage overlap and particle trapping on non-plate surfaces, which limits the

accuracy of the particle size distribution. The collection efficiency of each stage must therefore be calibrated.

Field Flow Fractionation

- Size Range: 1nm – 100um; typical concentration: > ppt; particle mass measurement; equivalent spherical hydrodynamic diameter

Field flow fractionation (FFF) is a particle classification/separation technique that takes advantage of the well characterized velocity profile of fluids in thin channels under laminar flow. By applying a field perpendicular to the direction of flow, particles of different properties are separated based on their interaction with the field resulting in differential positioning across the channel and therefore differences in elution times. For particle size applications the applied field is typically gravitational or cross-flow [i.e., as in asymmetric field flow fractionation (AFFF)] in nature. The applicable size range for commercial gravitational systems is typically 7nm to 20microns, and from 1nm to 100 microns for commercial cross-flow systems, but the dynamical range within a single device setting rarely exceed one order of size magnitude. Detection of the eluted materials can be carried out by a number of methods including UV absorbance, differential RI, multi-angle light scattering (MALS), dynamic light scattering (DLS) amongst others. Fractions can also be collected for analysis via microscopy and other techniques such as small angle x-ray scattering. An equivalent hydrodynamic sphere based particle size is calculated from the FFF theory based on elution time. This size can subsequently be compared to the particle size determined via light scattering and other methods. Major errors that occur with this method typically involve membrane/wall interactions and swelling as well as agglomeration that could occur in the channel.

Analytical Ultracentrifugation (AUC) and Centrifugal Liquid Sedimentation (CLS)

- Size range: 1nm – Several microns; typical concentration depends on method and material, up to several mass percent, equivalent spherical hydrodynamic diameter

In the shortest description, the analytical centrifuge uses synchronized detection system (turbidity, X-ray, schlieren, UV-VIS, interference) to monitor a colloidal system during its fractionation by centrifugal forces. The different commercially available geometries, disc (CPS, Brookhaven) vs. cuvette (LUM, Beckman) do not compromise the comparability of results. Standards were established for centrifuges (ISO 2001) with turbidity optics (ISO 2007b) and for those with X-ray absorption optics (ISO 2004). The commercial programs of turbidity centrifuges by Brookhaven and CPS, but also the programs for the turbidity optics integrated in Beckman centrifuges perform an iterative evaluation: first, one gets the diameter information from the measured time when the transmitted light intensity changes during constant or increasing speed of rotation (sedimentation rate); then one inverts the respective turbidity signal for each of these diameters via Mie theory to the mass-weighted size distribution. The viscosity of the dispersing medium and density of the solvent and the particle are required input parameters. X-ray and interference detection are especially useful for nano-classification (Wohlleben 2012), because these detection methods read directly the mass-% of each fraction, without significant conversion artifacts (e.g., Mie scattering applied in DLS) or assumption. Available Standards: ISO 13317-1:2001 (ISO 2001), ISO 13318-3:2004 (ISO 2004), ISO 13318-2:2007 (ISO 2007).

Hydrodynamic Chromatography

Hydrodynamic Chromatography (HDC) in a of a packed column fractionating method for particulate systems has been introduced, investigated and baptized in 1974 by Hamish Small

from The Dow Chemical Company. HDC specializes on “the discovery that the rate of transport of colloidal sized particles through a bed packed with solid, non-porous particles depends both on the particle size of the colloid and of the particles that constitute the packing” (Small 1974). The separation column is made of non-porous cross-linked polystyrene beads with narrow size distribution around 15 μm, enabling a working range of 10nm – 1200nm. Both diameter and peak width are deconvoluted with respect to calibration measurements, including Mie correction of shares derived from the UV-extinction at 254 nm. Particle size analysis by this method has recently been reviewed (Wohlleben and Schuch 2010).

Dynamic Mobility Analysis (DMA) and Electrospray-Dynamic Mobility Analysis (ES-DMA) (see Non-Imaging Counting Techniques)

Ensemble Techniques

Ensemble particle sizing techniques analyze a field of particles at a time. These methods typically employ curve fitting routines and are largely insensitive to low mass fraction particle content. The methods below are not recommended for classification purposes unless desired under the optional internal screen.

Small Angle X-ray/Neutron Scattering.

- Size Range: 1 nm to over 150 nm; typical concentration: > ppt; scattering cross-section measurement; equivalent scattering spherical diameter

Small angle X-ray (SAXS) and neutron (SANS) scattering involves the measurement and interpretation of elastically scattered radiation (i.e., X-rays or Neutrons) by a sample and the resulting scattering pattern is analyzed to provide information about the size, shape, and

orientation of particles within the samples. With minor adjustments to account for the different types of radiation, the same basic equations and “laws” (e.g., those due to Guinier, Zimm, Kratky, and Porod) can be used to analyze the scattering data. SAXS and SANS are highly sensitive to the data analysis routines and curve fitting applied especially for polydisperse and complex particle systems. However, these methods have the benefit that they provide the primary particle size information in addition to agglomerate/aggregate size distributions. SANS remains a technique that is only available at National Laboratory facilities; however, commercial laboratory SAXS systems are available.

Ultrasonic Attenuation Spectroscopy

- Size Range: 10nm – 10microns; typical concentration: >1 vol%; acoustic scattering cross-section; equivalent scattering spherical diameter

Ultrasonic Attenuation Spectroscopy is based on analyzing the attenuation of the amplitude of sound waves over a range of frequencies (typically 1MHz to 160 MHz) as they are transmitted through a measurement chamber containing a dispersion. The attenuation process involves scattering, diffraction, viscous, and thermal losses that collectively can be modeled through fundamental equations base on the conservation of mass, energy, and momentum. In this manner, the attenuation spectrum associated with any particle size distribution and particle concentration can be predicted for any suspension as long as a set of mechanical, thermodynamic and transport properties is known for both the dispersed and continuous phase. Unfortunately, the inverse problem is more difficult due to the potential of multiple solutions in complex systems. Significant errors and issues also arise in the analysis of irregular, porous, and polydispersed materials. Available Standards: ISO 20998-1:2006 (ISO 2006), ISO 20998-2:2013 (ISO 2013b).

Laser diffraction spectroscopy

Laser diffraction spectroscopy is the most widely used measurement technique for particles larger than one micrometer. In laser diffraction spectroscopy a beam of monochromatic light, usually a laser beam, is passed through the dispersed sample. The particles to be measured might be liquid or solid, the continuous phase might be gas or liquid. The concentration of the disperse phase must be adjusted to be adequate for the technique. For small particles this concentration can be lower than 0.002% in volume. The light scattered by the particles is detected by a multi-element detector. The measured light intensities are then transformed using optical models and mathematical algorithms to a volumetric particle size distribution. The method is considered an ensemble technique since the measured scattering intensities origin from an ensemble of particles which are illuminated simultaneously. When particle size decreases to or below the wave length of the monochromatic light to optical models used become strongly dependent on the complex refractive index of the particles and the algorithms used for deconvolution. Accuracy and resolution usually also decreases for nanoparticles. Details of the method are described in ISO 13320:2009 (ISO 2009). Laser diffraction spectroscopy should be regarded as a supplementary method with respect to the EU Nano definition. It is best suited to get an idea of the fraction of particles above 0.1 μm.

Dynamic Light Scattering (DLS) and Similar Techniques

DLS, [also known as quasi elastic light scattering (QELS), and photon correlation spectroscopy (PCS)] is suitable for the characterization of diluted colloidal particles over a wide range of sizes from a few nanometers to several micrometers. During a DLS measurement, the intensity fluctuations of light scattered from dispersion are analyzed by calculating of the time correlation function of the photon count rate as a function of the correlation delay time. From correlation

function it is possible to calculate Brownian diffusion coefficient. In the next step diffusion coefficient is transformed into intensity averaged hydrodynamic particle size (cumulant algorithm) using the viscosity of the liquid as an input parameter. The particle size distribution is derived from a deconvolution of the measured intensity autocorrelation function (e.g., CONTIN algorithm). In more sophisticated analysis Mie theory can be applied for the recalculation of intensity based distribution into number or volume based distribution. Only intensity averaged hydrodynamic particle size calculated via cumulant algorithm is accepted according to ISO standards.

2D or 3D cross-correlation and diffusive wave spectroscopy are variants of DLS that have been developed for the characterization of concentrated particle dispersions. These methods consider the coexistence of single and multiply scattered photons due to the higher particle concentrations; however, the application of these techniques does not provide a significant advantage over conventional dynamic light scattering for the particle sizing measurements on dispersions that can be diluted to a significant extent (i.e., solid particles). Available Standards: ASTM E2490-09 (ASTM 2009b), ISO 13321:1996 (ISO 1996), ISO 22412:2008 (ISO 2008).

References

Allen T. 1997. Particle size measurement. 5th ed. New York:Chapman & Hall.

Allmaier G, Laschober C, Szymanski WW. 2008. Nano es gemma and pdma, new tools for the analysis of nanobioparticles-protein complexes, lipoparticles, and viruses. J Am Soc Mass Spectrom 19:1062-1068.

ASTM International (ASTM). 2009a. ASTM Standard C690-09: Standard Test Method for Particle Size Distribution of Alumina or Quartz by Electric Sensing Zone Technique. Doi: 10.1520/C0690-09. Available: http://www.astm.org/Standards/C690.htm [accessed 11 September 2013].

ASTM International (ASTM). 2009b. ASTM Standard E2490-09: Standard Guide for the Measurement of Particle Size Distribution of Nanomaterials in Suspension by Photon Correlation Spectroscopy (PCS). Doi: 10.1520/E2490-09. Available: http://www.astm.org/Standards/E2490.htm [accessed 09 September 2013].

ASTM International (ASTM). 2011. ASTM Standard E2859-11: Standard Guide for Size Measurement of Nanoparticles Using Atomic Force Microscopy. Doi: 10.1520/E2859-11. Available: http://www.astm.org/Standards/E2859.htm [accessed 11 September 2013].

ASTM International (ASTM). 2012. ASTM Standard E2834-12: Standard Guide for Size Measurement of Particle Size Distribution in Suspension by Nanoparticle Tracking Analysis (NTA). Doi: 10.1520/E2834-12. Available: http://www.astm.org/Standards/E2834.htm [accessed 11 September 2013].

Burg TP, Godin M, Knudsen SM, Shen W, Carlson G. Foster JS, et al. 2007. Weighing of Biomolecules, Single Cells, and Single Nanoparticels in Fluid. Nature 446 1066.

European Commission (EC). 2011a. Commission Recommendation of 18 October 2011 on the definition of nanomaterial (Text with EEA relevance). Official Journal of the European Union L 275: 38-40. Available: http://eur-lex.europa.eu/LexUriServ/LexUriServ.do?uri=OJ:L:2011:275:0038:0040:EN:PDF [accessed 09 September 2013].

Guha S, Pease LF, 3rd, Brorson KA, Tarlov MJ, Zachariah MR. 2011. Evaluation of electrospray differential mobility analysis for virus particle analysis: Potential applications for biomanufacturing. J Virol Methods 178:201-208.

Guha S, Li M, Tarlov MJ, Zachariah MR. 2012. Electrospray-differential mobility analysis of bionanoparticles. Trends Biotechnol 30:291-300.

Huang B, Bates M, Zhuang X. 2009. Super-resolution fluorescence microscopy. Annu Rev Biochem 78:993-1016.

International Organization for Standardization (ISO) 1996. ISO 13321:1996: Particle Size Analysis—Photon correlation spectroscopy. Available: http://www.iso.org/iso/home/store/catalogue_tc/catalogue_detail.htm?csnumber=21707 [accessed 11 September 2013].

International Organization for Standardization (ISO) . 2001. ISO 13318-1:2001. Determination of particle size distribution by centrifugal liquid sedimentation methods—Part 1: General Principles and Guidelines. Available: http://www.iso.org/iso/home/store/catalogue_tc/catalogue_detail.htm?csnumber=21704 [accessed 11 September 2013].

International Organization for Standardization (ISO) . 2004. ISO 13318-2:2004. Determination of particle size distribution by centrifugal liquid sedimentation methods—Part 3: Centrifugal Xray Method. Available: http://www.iso.org/iso/home/store/catalogue_tc/catalogue_detail.htm?csnumber=31503 [accessed 11 September 2013].

ISO (International Organization for Standardization). 2006. ISO 20998-1:2006 Measurement and characterization of particles by acoustic methods—Part 1: Concepts and procedures in ultrasonic attenuation spectroscopy. Available: http://www.iso.org/iso/home/store/catalogue_tc/catalogue_detail.htm?csnumber=43468 [accessed 12 September 2013].

International Organization for Standardization (ISO) 2007a. ISO 13319:2007(en): Determination of particle size distributions—Electrical sensing zone method. Available: http://www.iso.org/iso/home/store/catalogue_tc/catalogue_detail.htm?csnumber=42354 [accessed 11 September 2013].

International Organization for Standardization (ISO). 2007b. ISO 13318:2007: Determination of particle size distribution by centrifugal liquid sedimentation methods—Part 2: Photocentrifuge method. Available: http://www.iso.org/iso/home/store/catalogue_tc/catalogue_detail.htm?csnumber=45771 [accessed 11 September 2013].

International Organization for Standardization (ISO). 2008. ISO 22412:2008: Particle Size Analysis—Dynamic Light Scattering. Available: http://www.iso.org/iso/home/store/catalogue_tc/catalogue_detail.htm?csnumber=40942 [accessed 11 September 2013].

ISO (International Organization for Standardization). 2009a. ISO 15900:2009(en) Determination of particle size distribution—Differential electrical mobility analysis for aerosol particles. Available: https://www.iso.org/obp/ui/#iso:std:iso:15900:ed-1:v1:en [accessed 09 September 2013].

International Organization for Standardization (ISO) 2009b. ISO 13320:2009: Particle Size Analysis—Laser Diffraction. Available: http://www.iso.org/iso/home/store/catalogue_tc/catalogue_detail.htm?csnumber=44929 [accessed 11 September 2013].

International Organization for Standardization (ISO) 2010. ISO 9277:2010(en): Determination of the specific surface area of solids by gas adsorption. Available: http://www.iso.org/iso/home/store/catalogue_tc/catalogue_detail.htm?csnumber=44941 [accessed 09 September 2013].

ISO (International Organization for Standardization). 2013a. ISO/DIS 27891:2013 Aerosol particle number concentration – Calibration of condensation particle counters. (Draft International Standard) Available: http://www.iso.org/iso/home/store/catalogue_tc/catalogue_detail.htm?csnumber=44414 [accessed 09 September 2013].

ISO (International Organization for Standardization). 2013b. ISO 20998-2:2013 Measurement and characterization of particles by acoustic methods—Part 2: Guidelines for linear theory. Available: http://www.iso.org/iso/home/store/catalogue_tc/catalogue_detail.htm?csnumber=43468 [accessed **12** September 2013].

Rothe D, Wood S, Heeschen B. 2007. Imaging of submicron particulate in an optical flow cell. Microsc Micoranal 13:672-673.

Small H. 1974. Hydrodynamic chromatography a technique for size analysis of colloidal particles. J Colloid Interface Sci 48:147-161.

Vainrub A, Pustovyy O, Vodyanoy V. 2006. Resolution of 90 nm (lambda/5) in an optical transmission microscope with an annular condenser. Opt Lett 31:2855-2857.

Wohlleben W, Schuch H. 2010. Psd from hydrodynamic chromatography and comparison with other analytical techniques. In: Measurement of particle size distributions of polymer latexes, (Gugliotta L, Vega J, eds). Kerala, India:Research Signpost, 130-153.

Wohlleben W. 2012. Validity range of centrifuges for the regulation of nanomaterials: From classification to as-tested coronas. J Nanopart Res 14:1300.
